# Marmoset Viral Hepatic Inflammation Induced by Hepatitis C Virus Core Protein via IL-32

**DOI:** 10.3389/fcimb.2020.00135

**Published:** 2020-04-21

**Authors:** Bochao Liu, Xiaorui Ma, Qi Wang, Shengxue Luo, Ling Zhang, Wenjing Wang, Yongshui Fu, Jean-Pierre Allain, Chengyao Li, Tingting Li

**Affiliations:** ^1^Department of Transfusion Medicine, School of Laboratory Medicine and Biotechnology, Southern Medical University, Guangzhou, China; ^2^Guangzhou Blood Center, Guangzhou, China; ^3^Emeritus Professor of Transfusion Medicine, University of Cambridge, Cambridge, United Kingdom

**Keywords:** HCV core protein, viral hepatic inflammation, IL-32, PI3K pathway, common marmosets

## Abstract

Common marmosets infected with GB virus-B (GBV-B) chimeras containing hepatitis C virus (HCV) core and envelope proteins (CE1E2p7) developed more severe hepatitis than those infected with HCV envelope proteins (E1E2p7), suggesting that HCV core protein might be involved in the pathogenesis of viral hepatitis. The potential role of HCV core in hepatic inflammation was investigated. Six individual cDNA libraries of liver tissues from HCV CE1E2p7 or E1E2p7 chimera-infected marmosets (three animals per group) were constructed and sequenced. By differential expression gene analysis, 30 of 632 mRNA transcripts were correlated with the immune system process, which might be associated with hepatitis. A protein–protein interaction network was constituted by STRING database based on these 30 differentially expressed genes (DEGs), showing that IL-32 might play a central regulatory role in HCV core-related hepatitis. To investigate the effect of HCV core protein on IL-32 production, HCV core expressing and mock constructs were transfected into Huh7 cells. IL-32 mRNA and secretion protein were detected at significantly higher levels in cells expressing HCV core protein than in those without HCV core expression (*P* < 0.01 and *P* < 0.001, respectively). By KEGG enrichment analysis and using the specific signaling pathway inhibitor LY294002 for inhibition of PI3K, IL-32 expression was significantly reduced (*P* < 0.001). In conclusion, HCV core protein induces an increase of IL-32 expression via the PI3K pathway in hepatic cells, which played a major role in development of HCV-related severe hepatitis.

## Introduction

Hepatitis C virus (HCV), a serious infectious disease that is transmitted through blood, is one of the most common viral causes of liver disease, affecting more than 170 million people worldwide. Chronic HCV infection causes chronic viral hepatitis C and liver dysfunction, which relates to the progression of cirrhosis and human hepatocellular carcinoma (HCC) (Choo et al., 1991). HCV is a single-stranded RNA flavivirus and has a 9.6-kilobase genome (kb) that encodes 10 proteins: structural core and envelope E1, E2, and p7, non-structural NS2, NS3, NS4A, NS4B, NS5A, and NS5B, respectively. Some of these proteins could interact with host cellular factors and promote tumor growth *in vivo* and *in vitro* (Ray et al., 1996; Gale et al., 1999; Park et al., 2000; Lerat et al., 2002). Previous studies found that core, NS3, NS5A, and NS5B could affect cell proliferation (Machida et al., 2001; Massague, 2004; Hara et al., 2006) or enhance oncogenic transformation (Banerjee et al., 2010).

HCV core protein is involved in the regulation of liver cell proliferation and cell transformation. It is thought that HCV is an important factor leading to HCC, although the molecular mechanisms determining such functions of virus remained unclear. HCV core protein may interact with transcription factors of p53, p21, NF-kB, and 14-3-3 protein, which are known to be involved in the development of HCC (Banerjee et al., 2010). HCV core protein could inhibit apoptosis, which is mediated by TNF-α (Ray et al., 1998; Marusawa et al., 1999) and interacted with TNF receptor 1 and lymphotoxin-β receptor that is involved in apoptotic signaling (Marusawa et al., 1999). HCV core protein expression reportedly affected the cell cycle of hepatocytes (HepG2) by increasing the levels of cell-cycle-dependent kinase inhibitor (CdkI) p21 (Nguyen et al., 2003). Common marmosets (*Callithrix jacchus*) are a kind of New World small primates and can be infected by GB virus B (GBV-B), a flavivirus closer to HCV (Bukh et al., 1999). Marmosets infected with GBV-B exhibited typical viral hepatitis similar to hepatitis C patients (Lanford et al., 2003; Jacob et al., 2004) and could be used as a surrogate animal model for HCV infection (Bright et al., 2004). In order to explore the core protein function, the marmosets infected with chimeric viruses of HCV structural core and envelope protein (CE1E2p7) or envelope protein (E1E2p7) sequences integrated within GBV-B genome were comparatively analyzed in combination with previous infected animals (Li et al., 2014).

*De novo* transcriptome sequencing has been used widely for studying specific gene expression patterns in different tissues or at different developmental stages, prediction of new transcripts (Denoeud et al., 2008), identification of alternative splicing (Lin et al., 2016), detection of single-nucleotide polymorphisms (SNPs) (Trick et al., 2009), and discovery of insertions/deletions in transcripts (Trapnell et al., 2010). In this study, cDNA libraries of liver tissue samples from two groups of marmosets infected with HCV-CE1E2p7/GBV-B or HCV-E1E2p7/GBV-B chimeras were sequenced. The IL-32 expression induced by HCV core protein was identified, which was demonstrated to play a critical role in occurrence of hepatic inflammation during HCV infection.

## Materials and Methods

### Ethics Statement

The use of common marmoset experimentation was approved by the Southern Medical University (SMU) Animal Care and Use Committee (permit numbers: SYXK[Yue]2010-0056). All animal care and procedures (NFYYLASOP-037) were in accordance with national and institutional policies for animal health and well-being. All efforts were made to minimize suffering of animals.

### Animal Liver Tissue Samples

Six common marmosets (*C. jacchus*) were obtained from Tianjin Medical University and individually fed in Laboratory Animal Research Center of Nanfang Hospital, Guangzhou, China. Liver tissue samples were collected specifically for this study from the animals infected with HCV/GBV-B chimeras in our previous study (Li et al., 2014).

### Histopathological Examination

Small sections of liver tissue from left, right, and caudate lobes of each animal liver were examined with hematoxylin and eosin (H&E) staining as described previously (Li et al., 2014). The necrosis and inflammation were graded on a 0–18 scale according to the modified HAI system (Knodell et al., 1981).

### CDNA Libraries and Sequencing

Total RNA was isolated from liver tissue samples using TRIzol reagents according to the manufacturer's introduction (Invitrogen, Carlsbad, USA). Hepatic mRNAs were isolated from the extracted total RNA by Oligo (dT) after treatment with DNase I and then reversely transcribed to cDNAs. The purified cDNA fragments were connected with adapters and the suitable fragments were amplified by PCR. The quality control (QC) was implemented in cDNA library establishment by using Agilent 2100 Bioanalyzer and ABI StepOnePlus Real-Time PCR System. The cDNA libraries were sequenced by using Illumina HiSeq4000.

### Sequence Data Processing and Analysis

In order to get clean sequencing data, the raw sequence reads were filtered for the low-quality sequences by eliminating the adaptors or a large amount of unknown sequencing reads. After mapping clean reads to reference genome, novel transcript prediction, SNP and INDEL detection, and differentially splicing gene (DSG) detection were performed. When obtaining novel transcripts, the coding sequences were compared with references to obtain a complete reference, and then gene expression analysis against this reference was performed. Differentially expressed genes (DEGs) were detected, in which possible function and pathway were analyzed by the Gene Ontology (GO) annotation system and the KEGG database, respectively. GO terms or pathways with a corrected *P* ≤ 0.05 were considered significantly enriched for DEGs. GO annotation results were analyzed by the Web Gene Ontology Annotation Plot (WEGO) software.

### Cells and Plasmids

Huh7 (human hepatocellular cell line) cells were cultured at 37°C in a 5% CO_2_ incubator in Dulbecco's modified Eagle medium (DMEM) supplemented with 10% FBS, 100 g/ml streptomycin, and 100 g/ml penicillin. The HCV or GBV-B core-expressing plasmid pcDNA3.1 constructs were generated by inserting either the full-length HCV core (genotype 1b) or the GBV-B core sequence as described previously (Li et al., 2014). T-vector containing the same sequence of HCV core or GBV-B core was used as non-expressing construct.

### Cell Transfection and Inhibition

A density of 4 × 10^5^ Huh7 cells in 2 ml of complete RPMI 1640 medium without antibiotics were plated in a 6-well plate for 24 h incubation. For transfection, 10 μl of Lipofectamine 2000 (Invitrogen) was diluted in 250 μl of FBS and antibiotic-free Opti-MEM (Invitrogen) and incubated for 5 min. Meanwhile, 2-μg DNA constructs (pcDNA3.1-HCV or -GBV-B core, T-vector-HCV or -GBV-B core) were diluted in 250 μl of Opti-MEM and incubated for 5 min. Then, the mixture of Lipofectamine and construct solutions and 1.5 ml of Opti-MEM were added onto the cells and incubated for 6 h. The medium was replaced with 10% FBS RPMI 1640 and incubated for 24–72 h for detection. Inhibition of signaling pathway in transfected Huh7 cells was conducted by incubation with the specific inhibitors (LY294002, SB203580, and SH-4-54) at different concentrations (5, 10, and 20 μM), respectively.

### Verification by Quantitative Real-Time RT-PCR

Total RNA of liver tissue samples or Huh7 cells were extracted using TRIzol method (Invitrogen) and reverse-transcripted using Reverse Transcription System according to the manufacturer's instructions (Roche, Basel, Switzerland). RT-PCR reactions were performed with SYBR Master Mix following the manufacturer's protocol (Roche, Basel, Switzerland). Amplification reactions were started for 2 min at 95°C, and then performed for 40 cycles at 95°C for 15 s, 55°C for 30 s, and 72°C for 30 s. All quantifications were carried out in triplicate and glyceraldehyde-3-phosphatedehydrogenase (GAPDH) was taken as internal control. Results were presented as mean value ± standard deviation (SD). IL-32-specific primers included a forward primer, 5′-CGACTTCAGAGAGTGCATGTT-3′, and a reverse primer, 5′-TGTTGCCTCTGAGTCGTAATTC-3′. The primers for the other analyzed proteins (IL-6, TNFa, IL18, IL8, IL1b, and GAPDH) were cited appropriately (Fujii et al., 2013; Jagessar et al., 2013). The fold change (FC) for the level of mRNA was calculated by the following equations: ΔCT = ΔCT(target) – ΔCT(GAPDH); ΔΔCT = ΔCT(infected) – ΔCT(control); mRNA fold change = 2–ΔΔCT.

### Immunohistochemistry

Immunohistochemical staining (IHC) was performed as previously described (Li et al., 2014). Briefly, after dewaxing and dehydration, the tissue slides were incubated with anti-IL-32 antibody (BioLegend, San Diego, CA). Then, the HRP-conjugated secondary antibody (PV-6002 Two-step IHC Detection Reagent, ZSGB company, Beijing, China) was added to the tissue sections and incubated for 30 min. Slides were developed with DAB and counterstained with hematoxylin, and then dehydrating in ethanol and xylene. The scoring was evaluated according to the intensity of staining and the frequency of stained cells (Koo et al., 2009).

### Enzyme Immunoassay (EIA)

The amount of IL-32 in culture supernatants was measured by a kit with Human IL-32 DuoSet ELISA (R&D Systems, Minneapolis, MN, USA).

### Immunofluorescence Staining (IFS)

Huh7 cells were seeded onto 24-well plates and transfected with pcDNA3.1-HCV core, pcDNA3.1-GBV-B core, or mock plasmid by Lipofectamine 2000 (Invitrogen, Guangzhou, China). The monoclonal antibody (mAb) to HCV core (C1F5 clone) or GBV-B core (1E5 clone) was used as primary antibody provided in the laboratory (Li et al., 2014), whereas Alexa Fluor 594 (red) goat anti-mouse IgG (Invitrogen) was used as secondary antibody for detection of the core protein in transfected cells. Diamidinophenylindoldiacetate (DAPI) was added to stain cell nuclei.

### Western Blot

Cells were lysed with RIPA buffer containing protease inhibitors at 24–72 h after transfection or treatment, and total protein lysate of each group was separated by 12% SDS-PAGE, and then transferred to a PVDF membrane (Millipore). The membranes were saturated with blocking solution (containing 1% BSA) for 2 h at room temperature and then incubated with specific primary antibody overnight at 4°C. After washing with PBST, the membranes were incubated with HRP-conjugated secondary antibody for 1 h at room temperature. Immunostaining was detected using an ECL substrate and GAPDH served as the internal reference.

### Statistical Analysis

All experiments were performed at least three times independently. The data were analyzed using the statistical package SPSS v. 16.0. The results were presented as the mean ± SD. Difference between groups were analyzed by using Student's *t*-test, and *P* < 0.05 was considered statistically significant.

## Results

### Difference of Necroinflammatory Grade in Histopathological Changes of Liver Tissues Between Marmosets Infected by HCV Chimeras With or Without HCV Core Protein

**Figure 1:**
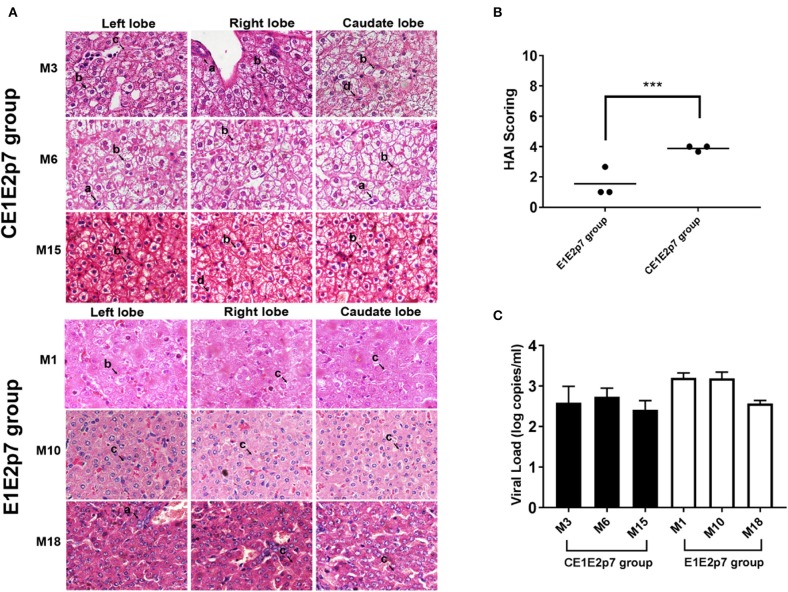
Difference of necroinflammatory grade in histopathological changes of liver tissues between marmosets infected by HCV chimeras with or without HCV core protein. **(A)** H&E staining was conducted on the liver tissues collected by left, right, and caudate lobes of each liver from marmosets. The original magnification was × 800. (a) Lymphocytic infiltrates, (b) ballooning degeneration (edema), (c) ground glass liver cells, and (d) eosinophilic cells. **(B)** Necroinflammatory grades in histopathological changes of liver tissues were scored by the modified HAI system, in which inflammation grades were on a scale of 0 to 18. ^***^P < 0.001. **(C)** The viral loads in the liver tissues infected with HCV-CE1E2p7/GBV-B or HCV-E1E2p7/GBV-B for the time points used for cDNA library construction and sequencing.

Among HCV/GBV-B chimera-infected marmoset models, we observed that the necroinflammatory grades of pathological changes in liver tissues from HCV-CE1E2p7/GBV-B chimera-infected marmosets (M3, M6, and M15) were obviously severe than those from marmosets (M1, M10, and M18) infected by HCV-E1E2p7/GBV-B chimera without HCV core protein. To further confirm this phenomenon, the liver tissue sections from left, right, and caudate lobes of each animal liver were examined for histopathological changes (Figure 1A). The HAI scores were evaluated for significant difference between two chimeric virus-infected marmosets with HCV core or without HCV core protein (mean of HAI score: 3.89 vs. 1.56; Table 1 and Figure 1B, *P* < 0.001). The viral loads in the liver tissues at the time points used for cDNA library construction and sequencing are shown in Figure 1C. The data suggested that HCV core might play a role in leading to severely hepatic inflammation of HCV chimera-infected marmosets.

**Table 1:** Histopathological observations.

**Virus**	**Marmoset**	**Time point (week)**	Necroinflammatory grade^*^			
			**Left lobe**	**Right lobe**	**Caudate lobe**	**Mean value**
CE1E2p7	M3	26	3	4	4	3.67
	M6	20	4	4	4	4
	M15	44	4	4	4	4
E1E2p7	M1	37	4	2	2	2.67
	M10	29	1	1	1	1
	M18	17	1	1	1	1

* *The histological status was determined by the modified HAI system (Kondell score), which grades necrosis and inflammation on a scale of 0–18 (periportal inflammation and necrosis, 0–10; lobular inflammation and necrosis, 0–4; portal inflammation, 0–4)*.

### *De novo* Assembly of Illumina Sequencing Reads and Annotation of DEGs

To reveal the difference of genomic transcripts in liver tissues between HCV chimera-infected marmosets with or without HCV core protein, six cDNA libraries of individual liver tissues from two groups of HCV-CE1E2p7 or -E1E2p7 chimera-infected marmosets were constructed and sequenced in a single run. After mapping the sequence reads against reference genomes and the reconstructed transcripts, 26,758 novel transcripts were obtained, in which 17,413 were previously unknown. Splicing events for known genes generated 1,113 novel coding transcripts with unknown features, and the remaining 8,232 were long non-coding RNAs.

**Figure 2:**
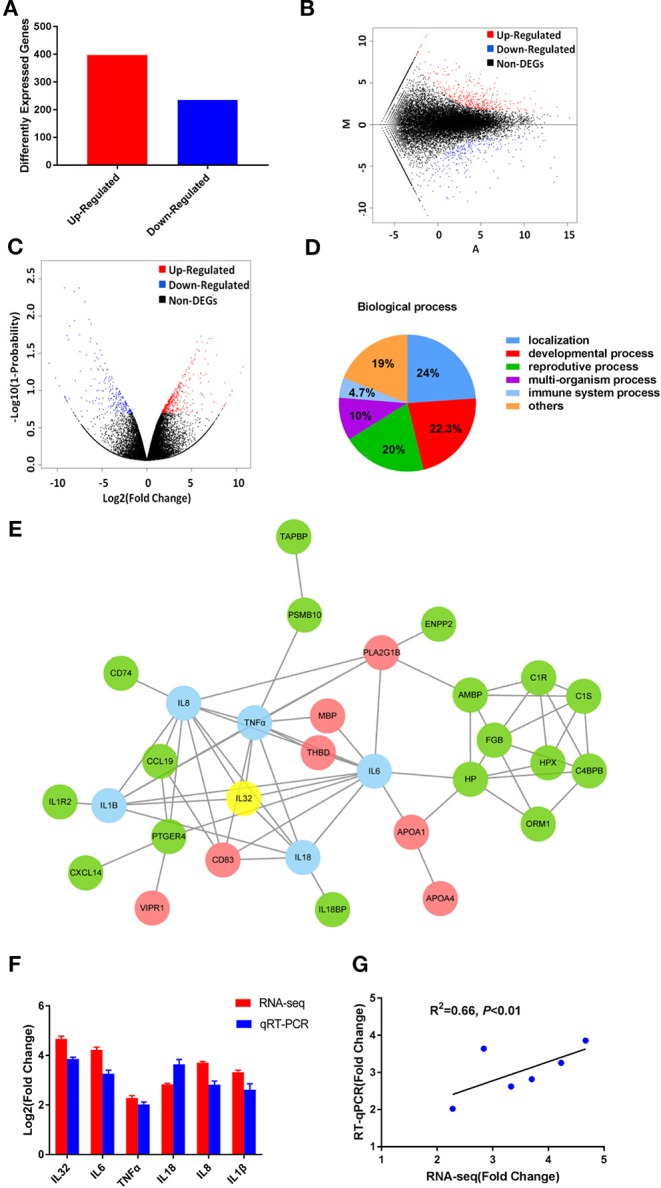
The RNA-seq results revealed the critical role of HCV core in the development of severe hepatic inflammation in HCV/GBV-B chimera-infected marmosets. **(A)** Among 632 DEGs, 235 genes were found to be down-regulated, while 397 genes were up-regulated in the group infected by CE1E2p7 chimera with HCV core protein. The MA plot **(B)** and the volcano plot **(C)** showed the distribution and significance of the differentially expressed genes (DEGs). **(D)** Among 632 DEGs, 30 genes were correlated with immune system process, which were contained in the biological process when performing GO classification and functional enrichment. **(E)** IL-32 played a core regulatory role in protein–protein interaction network constituted by the 30 DEGs involved in immune system process. The interaction network map was constructed by STRING database. Green indicated up-regulation, red indicated down-regulation, and blue indicated the DEGs correlated with IL-32, which was marked in yellow. The data were obtained from the transcriptome sequencing results. **(F)** The relative fold changes of IL-32 mRNA and the DEGs correlated with IL-32 in samples of marmoset's liver tissues were quantified by RT-qPCR and RNA-seq. Total RNA of liver tissue samples were extracted and reversely transcripted. All quantitative measurements were carried out in triplicate and normalized to GAPDH control in every reaction. Results were expressed as mean value ± standard deviation (SD) from three independent experiments. **(G)** Pearson correlation of fold changes (FC) in gene expression between RT-qPCR results and RNA-seq results (*P* < 0.01).

A transcript-level expression analysis was conducted to detect the differentially expressed mRNAs between two groups of HCV chimera-infected liver tissues using ballgown R package. Taking *P* < 0.05 and fold change (FC) > 1.5 as cutoff, 235 mRNAs were found to be down-regulated, while 397 mRNAs were up-regulated in the group infected by CE1E2p7 chimera with HCV core protein (Figure 2A). The MA plot (Figure 2B) and the volcano plot (Figure 2C) showed the distribution and significance of the differentially expressed genes (DEGs).

**Figure 3:**
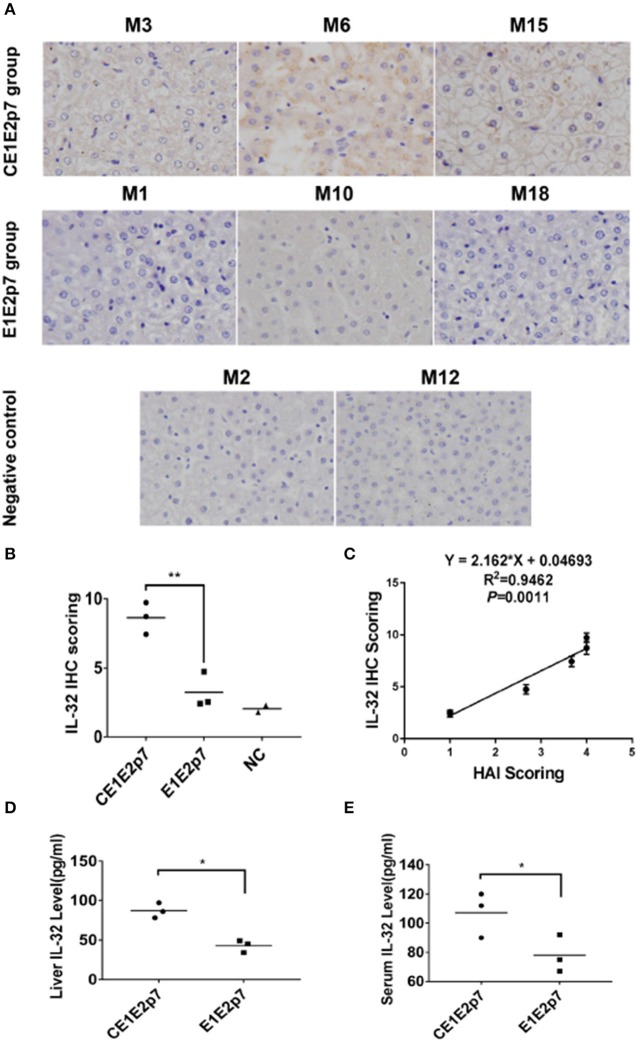
IL-32 protein was detected in liver tissues or sera from HCV chimera-infected marmosets. **(A)** Immunohistochemical staining of IL-32 protein in liver tissues from marmosets M3, M6, and M15 infected with HCV-CE1E2p7 chimera, or M1, M10, and M18 infected with HCV-E1E2p7 chimera. The original magnification was × 200. **(B)** The IHC score was assessed according to the intensity of staining (no staining, 0; weak staining, 1; moderate staining, 2; strong staining, 3) and the extent of stained cells (0%, 0; 1 to 10%, 1; 11 to 50%, 2; 51 to 80%, 3; 81 to 100%, 4). The final score was determined by multiplying the intensity scores by the extent of positivity scores of stained cells. The difference between HCV CE1E2p7 and E1E2p7 chimera-infected marmosets was significant (*P* < 0.01). **(C)** Level of IL-32 expression in liver tissues from six marmosets is correlated with necroinflammatory grades (HAI scores) in histopathological changes of liver tissues scored by the modified HAI. **(D)** Liver IL-32 protein or **(E)** serum IL-32 protein from six marmosets (the last week before sacrifice) was tested by ELISA. The difference between the two groups was significant (*P* < 0.05). Data were presented as mean value ± SD from three separate experiments. *P < 0.05, **P < 0.01.

Using DEGs, we performed GO classification and functional enrichment. Among 632 DEGs, 30 genes were correlated with immune system process, which played an important role in hepatitis and contained in biological process (Figure 2D). By comprehensive analysis of data obtained from these 30 DEGs, a protein–protein interaction network was constituted by STRING database (Figure 2E), which indicated that IL-32 played a core regulatory role in the immune system. To validate the RNA-seq results, the relative fold changes of IL-32 and the five DEGs including IL-6, TNF-α, IL-18, IL-8, and IL-1β that correlated with IL-32 in this network in liver tissue samples from HCV CE1E2p7 or E1E2p7 chimera-infected marmosets were measured by RT-qPCR (Figure 2F). The Pearson correlation of fold changes in gene expression between RT-qPCR and RNA-seq analysis was significant (Figure 2G), which suggested that RT-qPCR results were consistent with RNA-seq results. Immunohistochemical staining results showed that the relative mean density of hepatic IL-32 staining in liver tissues from HCV-CE1E2p7/GBV-B chimera-infected marmosets was significantly increased compared with that of animals infected with HCV-E1E2p7/GBV-B chimera (mean of IHC scores: 8.63 vs. 3.23; Figures 3A,B, *P* < 0.01). The levels of IL-32 expression (IHC scoring) in liver tissues were positively correlated with the HAI scores of histopathological changes in liver tissues from six marmosets (Figure 3C, *P* = 0.001, *R*^2^ = 0.9462), but not correlated with viral loads.

As shown in Figures 3D,E, level of hepatic or serum IL-32 from HCV-CE1E2p7/GBV-B chimera-infected marmosets was significantly higher than that from HCV-E1E2p7/GBV-B chimera-infected animals (*P* < 0.05).

### HCV Core Protein Induces IL-32 Production in Huh7 Cells

**Figure 4:**
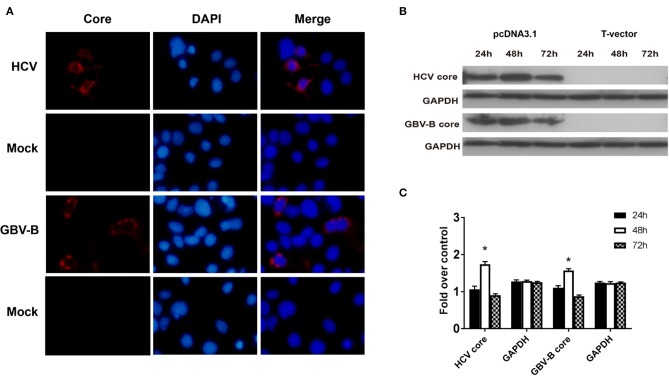
The expression of HCV core or GBV-B core protein was confirmed in the expressing vector transfected Huh7 cells. **(A)** Immunofluorescence staining for HCV or GBV-B core protein in transfected Huh7 cells. Huh7 cells were seeded onto 24-well plates and transfected with 1 μg of pcDNA3.1-HCV core or pcDNA3.1-GBV-B core or mock plasmid, respectively. After 48 h, the cells were stained with mAb to HCV core or GBV-B core. Cell nuclei were stained in blue with DAPI. Original magnification was ×200. **(B,C)** Western blot analysis for HCV core and GBV-B core proteins in transfected Huh7 cells at the time point of 24, 48, and 72 h. *P < 0.05 vs. the 24-h group. All experiments were repeated three times.

**Figure 5:**
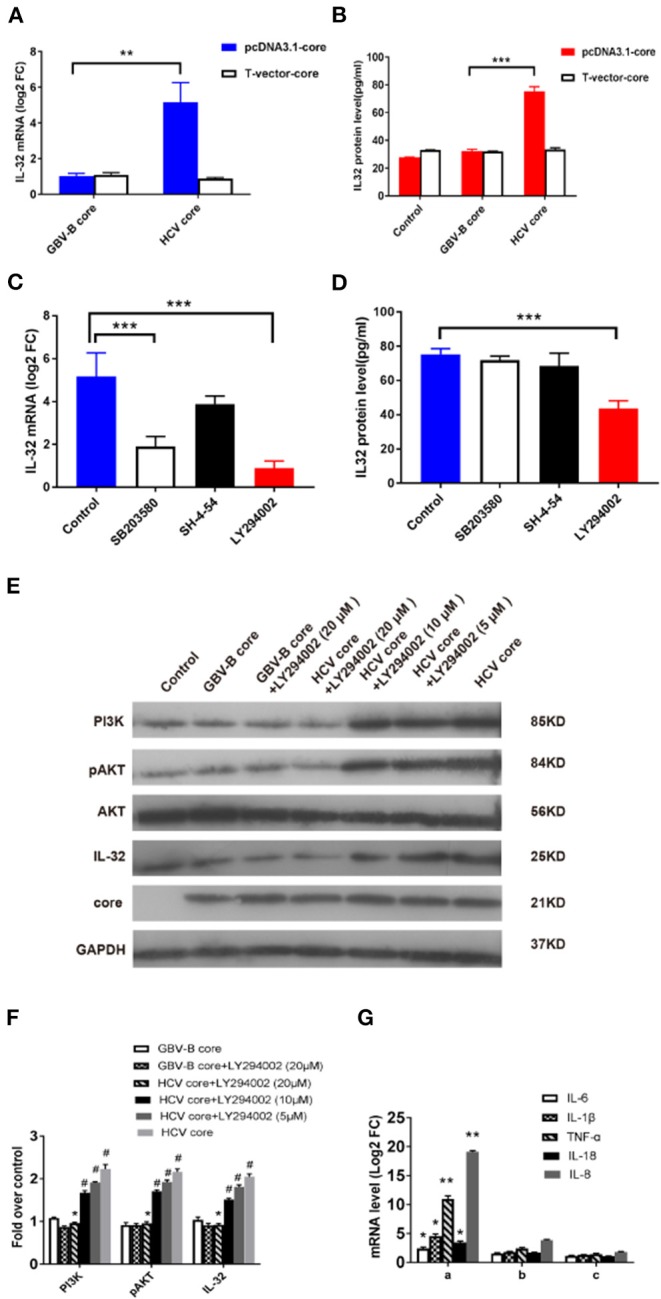
HCV core-induced IL-32 expression via the PI3K/AKT pathway. **(A)** The level of IL-32 mRNA (relative fold change, FC) in samples of Huh7 cells transfected for 48 h with pcDNA3.1-HCV core, pcDNA3.1-GBV-B core, or mock plasmid was quantified by RT-qPCR. **(B)** The level of IL-32 protein in culture supernatants of Huh7 cells in 48 h after transfecting with pcDNA3.1-HCV core, pcDNA3.1-GBV-B core, or mock plasmid was quantified by ELISA. **(C–E)** Inhibition of HCV core induced IL-32 expression in Huh7 cells by specific signaling pathway inhibitors. Huh7 cells were treated with the specific signaling pathway inhibitors of LY294002 (PI3K inhibitor), SB203580 (MAPK inhibitor), and SH-4-54 (STAT inhibitor), respectively, by following transfection with pcDNA3.1-HCV core construct DNA. After 48 h, **(C)** levels of IL-32 mRNA transcripts and **(D)** secretion protein were quantified by RT-qPCR and ELISA. Huh7 cells transfected with pcDNA3.1-HCV core but without inhibitor were used as negative control (NC). A reduction of IL-32 mRNA or protein in transfected cells was compared between inhibitor and control. **(E,F)** The levels of downstream molecules including PI3K, AKT, pAKT, and IL-32 were detected by Western blot for analyzing PI3K pathway. Huh7 cells transfected with mock plasmids were used as control. PI3K and IL-32 were normalized against GAPDH, and pAKT were normalized against GAPDH as well as total AKT. #*P* < 0.05 vs. control; *P < 0.05 vs. HCV core group; **P < 0.01; ***P < 0.001. **(G)** The level of inflammatory cytokine mRNAs from Huh7 cells in 48 h incubated with the (a) supernatants from pcDNA3.1-HCV core-transfected Huh7 cells; (b) supernatants from pcDNA3.1-HCV core-transfected Huh7 cells treated with 20 μM LY294002; (c) anti-IL-32 antibody neutralized supernatants from pcDNA3.1-HCV core-transfected Huh7 cells. The levels of inflammatory cytokine mRNAs from Huh7 cells in 48 h incubated with supernatants from mock plasmid-transfected Huh7 cells were set as controls. *P < 0.05 vs. control; **P < 0.01 vs. control. Data were presented as mean value ± SD from three separate experiments, in which each measurement was carried out in triplicate.

To investigate the effect of HCV core protein on IL-32 expression, pcDNA3.1-HCV core, pcDNA3.1-GBV-B core, and mock construct DNAs were transfected into Huh7 cells. Forty-eight hours later, the production of HCV core or GBV-B core protein was confirmed in the expressing vector-transfected cells but not in mock plasmid-transfected cells by immunofluorescence staining (Figure 4A) and Western blot (Figures 4B,C), suggesting that HCV core or GBV-B core protein was present in the cells, respectively. Meanwhile, IL-32 mRNA from transfected cells was measured by RT-qPCR (Figure 5A), showing that IL-32 mRNA level increased significantly in the cells transfected with HCV core expressing construct. It was five times higher than that in the cells transfected with GBV-B core expressing construct (*P* < 0.01).

To confirm the effect of HCV core protein on IL-32 expression, culture supernatants from transfected cells were tested for secreted IL-32 protein by ELISA. As shown in Figure 5B, IL-32 protein concentration was twofold higher in the supernatant of pcDNA3.1-HCV core construct-transfected cells than that in pcDNA3.1-GBV-B core-transfected cells (*P* < 0.001). The results suggested that the presence of HCV core protein could significantly increase IL-32 secretion in the supernatant of transfected Huh7 cells.

To eliminate the possibility that HCV core DNA might induce IL-32 expression, T-vector-HCV core, T-vector-GBV-B core, and empty-pcDNA3.1 or empty-T-vector mock plasmids were transfected into Huh7 cells. Forty-eight hours later, IL-32 at mRNA and supernatant protein levels from transfected Huh7 cells were measured by RT-qPCR and ELISA, respectively. The results showed no difference among T-vectorial HCV core DNA, GBV-B core DNA, and empty-vector controls (Figures 5A,B), suggesting that only HCV core protein could induce IL-32 expression in these cells.

### HCV Core-Induced IL-32 Expression via the PI3K/AKT Pathway

Since little was known about the regulation of IL-32 production, we further examined which signaling pathway was involved in production of IL-32 stimulated by HCV core protein in Huh7 cells. Here, 562 out of 632 DEGs were categorized to 268 KEGG pathways. To identify the most impacted pathways, a KEGG enrichment analysis was performed for HCV-CE1E2p7/GBV-B chimera-infected marmosets, which contained 92 pathways of *P* < 0.05. Among these pathways, PI3K/AKT, JAK/STAT, and MAPK were previously described relative to IL-32 (Nishida et al., 2008; Ko et al., 2011; Moschen et al., 2011).

To identify which pathway was involved in HCV core protein for inducing IL-32 production, the specific signaling pathway inhibitors of LY294002 (PI3K inhibitor), SB203580 (MAPK inhibitor), and SH-4-54 (STAT inhibitor) were utilized in pcDNA3.1-HCV core construct-transfected Huh7 cells. Levels of IL-32 mRNA transcripts and proteins were quantified by RT-qPCR and ELISA, respectively (Figures 5C,D). Approximately 60–80% reduction of IL-32 mRNA level in transfected cells was observed by PI3K and MAPK inhibitors (*P* < 0.001; Figure 5C), while a 46% reduction of IL-32 protein secretion in the supernatant of transfected cells was solely found by PI3K inhibitor (*P* < 0.001; Figure 5D). Furthermore, the levels of PI3K, pAKT, AKT, and IL-32 expression were quantified by Western blot (Figure 5E). When compared with the control group, HCV core protein increased the expression levels of PI3K, pAKT, and IL-32 in Huh7 cells. In contrast, when compared with the HCV core expression group, the inhibitor LY294002 (with concentrations of 5, 10, and 20 μM) inhibited the expression of PI3K, pAKT, and IL-32 in Huh7 cells in a dose-dependent fashion (Figure 5F). These results suggested that HCV core protein may induce IL-32 production via the PI3K/AKT pathway. Further, to identify the regulatory role of IL-32 associating with inflammatory cytokines, levels of mRNA transcripts for IL-6, IL-1β, TNFα, IL-18, and IL-8 were detected from Huh7 cells in 48 h incubated with the supernatants from pcDNA3.1-HCV core-transfected Huh7 cells only (Figure 5Ga), supernatants from transfected Huh7 cells treated with 20 μM LY294002 (Figure 5Gb), and anti-IL-32 antibody neutralized supernatants from transfected Huh7 cells (Figure 5Gc), respectively. Compared with the controls from mock plasmid-transfected Huh7 cells, these five inflammatory cytokine mRNAs were significantly elevated, especially 10.96-fold for TNFα and 19.13-fold for IL-8 (Figure 5Ga). After treating with LY294002 or anti-IL32 antibody, these mRNA levels decreased significantly (Figure 5Gb,c). These results suggested that IL-32 might act as a central role in the hepatic inflammation regulatory network.

## Discussion

Chronic HCV infection is a risk factor for development of hepatic steatosis, cirrhosis, and HCC (Raimondi et al., 2009). However, the exact molecular pathogenesis of chronic HCV infection-mediated hepatitis is not entirely explored. A study suggested that expression of the core protein increased cell proliferation, DNA synthesis, apoptosis, cell cycle progression, cell transformation, steatosis, and HCC in transgenic mice (Moriya et al., 1998). Looking back at our previously reported study (Li et al., 2014), we observed that the marmosets infected with HCV core protein-containing viral chimera experienced more severe hepatic inflammation than animals infected with viral chimera that did not express HCV core protein. These data suggested that HCV core might lead to hepatic inflammation. Despite the availability of complete genome sequence from marmosets (Worley et al., 2014), no data covers the transcriptome of liver tissue for marmosets. Based on the mRNA transcripts of liver tissues from HCV chimera-infected marmosets in the present study, we found that the presence of HCV core protein was positively correlated with the severe viral hepatitis in marmosets (Figure 3C), which might be a stage toward progression to HCC as demonstrated previously in mice for HCV core protein inducing HCC (Moriya et al., 1998).

In this study, the complexity of liver transcriptome of marmosets was analyzed by high-throughput RNA sequencing. We found 632 genes with expression patterns differentiating between two groups of marmosets infected with HCV CE1E2p7 chimera and E1E2p7 chimera, respectively. Among those mRNA transcripts, IL-32 was considered the most important factor leading to hepatitis in infected marmosets. IL-32 is a cytokine produced by T-cells, natural killer (NK) cells, monocyte/macrophage, and epithelial cells, that includes six isoforms of IL-32α, β, γ, δ, ε, and ζ. IL-32α is the most abundant isoform (Ko et al., 2011). IL-32 could activate the NF-kB and p38-MAPK pathways to induce proinflammatory cytokines (IL-1β, IL-6, and TNFα) and chemokines (IL-8 and MIP-2) by stimulating monocytes and macrophages (Yousif et al., 2013). A previous study indicated that the levels of IL-32 mRNA were significantly correlated with hepatic inflammation and that HCV infection of Huh 7.5 cells increases IL-32 expression (Moschen et al., 2011). However, this study indicated that HCV infection induced IL-32 transcription in Huh7.5 cells without clarifying whether the IL-32 expression was induced by HCV core protein. Further, to confirm IL-32 relating to the hepatic inflammation regulatory network, five inflammatory cytokine mRNAs (IL-6, IL-1β, TNFα, IL-18, and IL-8) were measured from Huh7 cells incubated with or without the functional IL-32 (Figure 5G). The data supported the idea that IL-32 promoted the production of inflammatory cytokines. To test the hypothesis that HCV core protein induced IL-32 expression, pcDNA3.1-HCV core or -GBV-B core expressing constructs, pT-Vector-HCV core or -GBV-B core non-expressing constructs, and empty-vector controls were transfected into Huh7 cells. We first found that HCV core protein expression induced an increase of IL-32 mRNA transcripts and secretion proteins in transfected cells. Since IL-32 exerts pro-inflammatory effects in various cell types including epithelial, endothelial, and mononuclear cells (Kim et al., 2005; Nold-Petry et al., 2009), our results might explain the important role of HCV core protein in developing viral hepatitis.

As described previously, the PI3K/AKT pathway regulates IL-32α production in human alveolar epithelial cells (Ko et al., 2011) and also mediates IL-32α induction in human pancreatic periacinar myofibroblasts (Nishida et al., 2008). MAPK signaling pathway is also related to MyD88-dependent IL-32α production in IL-1β-stimulated human alveolar epithelial cells (Moschen et al., 2011). TNFα alone or in combination with IFNα could induce IL-32 production in Huh7.5 cells as well as in Hep3B cells. The IL-32 induction was completely abrogated by inhibition of NF-kB signaling (by BAY11-7082) but not JAK/STAT signaling (by Jak Inhibitor I). In contrast to hepatocytes, IL32 induction was dependent on both NF-kB and JAK/STAT signaling pathways in CD14^+^ monocytes (Moschen et al., 2011). Since the NF-kB pathway was not found in the 92 pathways obtained from DEG sequencing results (*P* < 0.05), we focused on PI3K/AKT, JAK/STAT, and MAPK as the potential targets for the pathways involved in IL-32 induction stimulated by HCV core protein. To explore the critical role of those signaling pathways, specific signaling pathway inhibitors of PI3K (LY294002), MAPK (SB203580), or STAT inhibitor (SH-4-54) were added to HCV core expressing construct-transfected Huh7 cells. A reduction of IL-32 mRNA level in transfected cells was obtained by PI3K and MAPK inhibitors, while only a reduction of secretion IL-32 protein was identified in the supernatant of transfected cells by PI3K inhibitor. This suggested that HCV core protein-induced IL-32 production was mainly through the PI3K pathway. Western blot results show that (Figures 5E,F) HCV core protein increased the expression of PI3K, pAKT, and IL-32 in Huh7 cells. In contrast, LY294002 inhibited the expressions of PI3K, pAKT, and IL-32 in Huh7 cells. Thus, the changes in the expression levels of IL-32 in HCV core construct-transfected Huh7 cells were regulated by the PI3K/AKT signaling pathway.

The secretion of IL-32α could be stimulated by IL-1β in A549 cells, regulated by the PI3K/AKT signaling pathway, and suppressed by inhibitors of SFKs, PKCδ, or p38 (Ko et al., 2011). IL-32 could also be constitutively produced in Huh7.5 cells stimulated by IL-1β and TNFα (Moschen et al., 2011), and it also could be induced in monocyte by HCV (Pang et al., 2016). IL-1β acts an important role in various cellular responses such as inflammation (Charles, 1997). The binding of IL-1β to type I IL-1 receptors (IL-1RI) could trigger recruitment of the adapter protein MyD88, which could affect PI3K/AKT signaling pathway by regulating PKCδ and PI3K (Braddock and Quinn, 2004). HCV could activate production of IL-1β through the NLRP3 inflammasome pathway (Ramos et al., 2012), which could show response in both acute and chronic inflammation (Allen et al., 2009). The exact mechanism by which HCV core protein stimulates IL-32 expression and secretion through the PI3K/AKT pathway may be dependent on inflammatory cytokines like IL-1β or TNFα. As the relationship between HCV core and IL-1β (or TNFα) was not explored in this study, we focused on how HCV core protein affected PI3K/AKT signaling pathway, and we would examine that if IL-1β or TNFα was involved in this progression in the future.

In summary, an increase of IL-32 production was induced by HCV core protein via the PI3K/AKT pathway, which might explain the association with a high grade of hepatic inflammation in HCV-infected individuals. Clearance of HCV core protein or modulation of IL-32 activity might be an option to reduce inflammation in patients with chronic hepatitis C.

## Data Availability Statement

All data associated with this study are available in the main text or the Supplementary Materials, and HCV/GBV-B chimeras or constructs are available in the Department of Transfusion Medicine, Southern Medical University, Guangzhou, China.

## Ethics Statement

The animal study was reviewed and approved by Southern Medical University (SMU) Animal Care and Use Committee (permit numbers: SYXK(Yue)2010-0056).

## Author Contributions

CL and TL designed research. BL, XM, QW, SL, LZ, WW, and TL performed research. CL, BL, TL, and YF analyzed data. BL, TL, CL, and J-PA wrote the paper.

## Conflict of Interest

The authors declare that the research was conducted in the absence of any commercial or financial relationships that could be construed as a potential conflict of interest.
